# One Step Forward with Dry Surface Biofilm (DSB) of *Staphylococcus aureus*: TMT-Based Quantitative Proteomic Analysis Reveals Proteomic Shifts between DSB and Hydrated Biofilm

**DOI:** 10.3390/ijms232012238

**Published:** 2022-10-13

**Authors:** Md. Arifur Rahman, Ardeshir Amirkhani, Farhana Parvin, Durdana Chowdhury, Mark P. Molloy, Anand Kumar Deva, Karen Vickery, Honghua Hu

**Affiliations:** 1 Surgical Infection Research Group, Faculty of Medicine, Health and Human Sciences, Macquarie University, Sydney 2109, Australia; 2 Haematology Clinical Trials Unit, Liverpool Hospital, Sydney 2170, Australia; 3 Australian Proteome Analysis Facility, Macquarie University, Sydney 2109, Australia

**Keywords:** *Staphylococcus aureus*, biofilms, proteome, TMT, mass spectrometry, dry surface biofilms, metabolic processes, biosynthetic processes, transport systems, stress responses

## Abstract

The Gram-positive bacterium *Staphylococcus aureus* is responsible for serious acute and chronic infections worldwide and is well-known for its biofilm formation ability. Recent findings of biofilms on dry hospital surfaces emphasise the failures in current cleaning practices and disinfection and the difficulty in removing these dry surface biofilms (DSBs). Many aspects of the formation of complex DSB biology on environmental surfaces in healthcare settings remains limited. In the present study, we aimed to determine how the protein component varied between DSBs and traditional hydrated biofilm. To do this, biofilms were grown in tryptic soy broth (TSB) on removable polycarbonate coupons in the CDC biofilm reactor over 12 days. Hydrated biofilm (50% TSB for 48 h, the media was then changed every 48 h with 20% TSB, at 37 °C with 130 rpm). DSB biofilm was produced in 5% TSB for 48 h at 35 °C followed by extended periods of dehydration (48, 66, 42 and 66 h at room temperature) interspersed with 6 h of 5% TSB at 35 °C. Then, we constructed a comprehensive reference map of 12-day DSB and 12-day hydrated biofilm associated proteins of *S. aureus* using a high-throughput tandem mass tag (TMT)-based mass spectrometry. Further pathway analysis of significantly differentially expressed identified proteins revealed that proteins significantly upregulated in 12-day DSB include PTS glucose transporter subunit IIBC (PtaA), UDP-N-acetylmuramate-L-alanine ligase (MurC) and UDP-N-acetylenolpyruvoylglucosamine (MurB) compared to 12-day hydrated biofilm. These three proteins are all linked with peptidoglycan biosynthesis pathway and are responsible for cell-wall formation and thicker EPS matrix deposition. Increased cell-wall formation may contribute to the persistence of DSB on dry surfaces. In contrast, proteins associated with energy metabolisms such as phosphoribosyl transferase (PyrR), glucosamine--fructose-6-phosphate aminotransferase (GlmS), galactose-6-phosphate isomerase (LacA), and argininosuccinate synthase (ArgG) were significantly upregulated whereas ribosomal and ABC transporters were significantly downregulated in the 12-day hydrated biofilm compared to DSB. However, validation by qPCR analysis showed that the levels of gene expression identified were only partially in line with our TMT-MS quantitation analysis. For the first time, a TMT-based proteomics study with DSB has shed novel insights and provided a basis for the identification and study of significant pathways vital for biofilm biology in this reference microorganism.

## 1. Introduction

*Staphylococcus aureus* frequently causes healthcare-associated infections (HAI) worldwide, and its virulence is increased due to its ability to form biofilm. Biofilms are problematic in healthcare settings, accounting for 65% of HAI including indwelling medical devices such as prostheses, peripheral venous catheters and urinary catheters. In addition, biofilms have been found to contaminate heat-sensitive surgical equipment such as endoscopes [1] but also instruments that are subjected to heat sterilization [2]. Environmental surfaces in healthcare facilities readily become contaminated with pathogens and these play a vital role in terms of disease transmission [3,4,5]. The risk of pathogen transmission is reduced by environmental cleaning and disinfection [6,7]; however, biofilm formation on the surfaces in these settings complicates environmental decontamination as biofilms are more difficult to remove by cleaning agents and are more tolerant of disinfectants [8,9,10,11,12,13]. They, therefore, can function as reservoirs of pathogens in the hospital setting and provide favourable environments for the long-term persistence of pathogens [8,14,15,16].

Dry surface biofilms (DSB) form on dry surfaces and are subjected to osmotic stress. DSB can survive for over 12 months without nutrition or exposure to hydrating media [8]. In contrast, traditional hydrated biofilm form in a fluid environment and are thus not subjected to osmotic stress. As more than 50% of DSB recovered from hospital surfaces contain *S. aureus* [8,17], we developed a novel *S. aureus* DSB model [18] which was similar in chemical makeup to DSB found on dry hospital surfaces. To model DSB, the nutrient supply is reduced to minimal levels, the biofilm is subjected to water stress interspersed at room temperature (RT, 22–25 °C) with short periods of nutrition over 12 days. We want to know how the bacteria adapt and continue to grow under these harsh conditions.

DSB contains more proteinaceous components, less carbohydrate, and has thicker extracellular polymeric substances (EPS) than hydrated biofilm [18]. In *Staphylococcus* biofilms, proteins such as fibronectin-binding proteins (FnBPs) and biofilm-associated proteins (Bap), polysaccharide intercellular adhesion (PIA), extracellular DNA (eDNA), and teichoic acids are components of EPS matrix [19].

Recent advances in isobaric multiplexed labelling techniques have considerably enhanced the depth, effectiveness, and performance of MS-based proteomics. In TMTs labelling, each isotopic variant contains unique reporter masses but has the same overall mass, allowing precise general quantification along with accurate measurement of the relative abundance of proteins between samples even for low mass proteins [20,21]. TMT labelling permits multiplexing of up to ten samples simultaneously [22,23,24], thus saving instrument time, and simplifying the experimental design [25], yet can identify several thousand non-redundant proteins simultaneously [23,26,27].

There is a dire need to understand the deeper biology of complex biofilm so that cleaning agents and disinfectants can be designed to destabilise and remove the biofilm. The present study used a novel strategy, a high-throughput TMT-based mass spectrometry (MS), to examine the changes in protein expression profiles by comparing the proteomic profiles of *S. aureus* biofilm grown for 12 days either in the absence of osmotic stress and therefore fully hydrated (12-day biofilm) or; subjected to periodic dehydration and osmotic stress (DSB) with one-day planktonic culture. This new strategy and a thorough analysis of proteomics profiles facilitated gaining new knowledge on the protein composition of both hydrated biofilm and DSB and an understanding of biofilm regulators and the probable mechanisms for the formation of *S. aureus* biofilms in these modes of growth.

## 2. Results and Discussion

### 2.1. TMT Identification of Differentially Regulated Proteins in the Hydrated Biofilm and DSB

**Figure 1:**
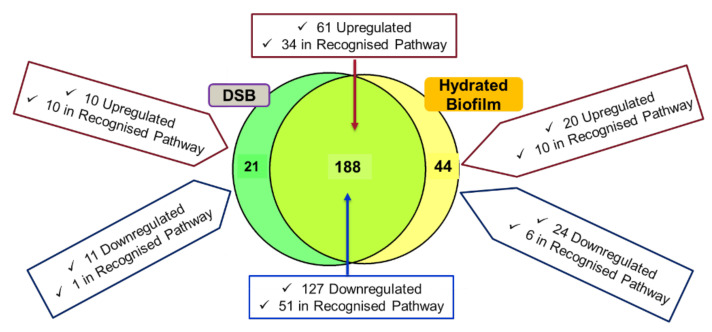
This Venn diagram represents identified proteins dysregulated >2-fold, *p* < 0.05 in comparison with 24 h planktonic culture. It shows the dispersion of common proteins (188) between hydrated and DSB as well as uniquely identified proteins based on abundance ratios in the hydrated biofilm (44) and DSB (21). Pathway analysis was performed using KEGG.

In this study, *S. aureus* reference strain was grown in tryptic soy broth to produce a 24 h planktonic culture, and over 12 days in the CDC biofilm reactor to produce the hydrated biofilm and dry surface biofilm. Then, we performed in-solution digestion followed by TMT-based high-resolution mass spectrometry and investigated what changes occur in the proteome of hydrated biofilm and dry surface biofilm in comparison with 24 h planktonic. A total of 1636 non-redundant proteins with at least one unique peptide and <1% FDR were identified and quantitated. However, in this study we focused on proteins dysregulated > 2-fold, *p* < 0.05, to make the changes more biologically relevant. Of these, 20 and 10 proteins were significantly (>2-fold) upregulated compared to planktonic bacteria in hydrated biofilm and DSB, respectively (Figure 1). Of which, 10 proteins were detected in recognised protein pathways in hydrated biofilm and DSB (Figure 1). In contrast, 24 and 11 proteins were significantly (>2-fold) downregulated in hydrated biofilm and DSB, respectively. Of which, six and one protein were detected in recognised protein pathways in hydrated biofilm and DSB, respectively (Figure 1).

Among the proteins upregulated in both 12-day hydrated biofilm and DSB, 61 proteins were significantly upregulated (>2-fold) compared to planktonic cultures. Of these proteins, 34 were involved in recognised pathways. There were 127 proteins significantly downregulated (>2-fold) in both biofilms of which 51 proteins were involved in recognised pathways (Figure 1). Among them, purine and pyrimidine metabolism, energy metabolism, and amino acid biosynthesis-associated pathways were identified in upregulated protein clusters, whereas translation, pyruvate metabolism, energy metabolism, and amino acid biosynthesis-associated pathways were downregulated. In the hydrated biofilm dysregulated proteins are more upregulated (Table S1 in Supplementary Materials) and less downregulated (Table S2 in Supplementary Materials) than the same proteins in DSB.

### 2.2. Energy Metabolism-Associated Proteins Are Significantly Upregulated in the Hydrated Biofilm

The proteins that are significantly upregulated in the hydrated biofilm in comparison with DSB are listed in Table 1. In addition, the full list of significantly upregulated proteins in the hydrated biofilm is available in Table S3.

**Figure 2:**
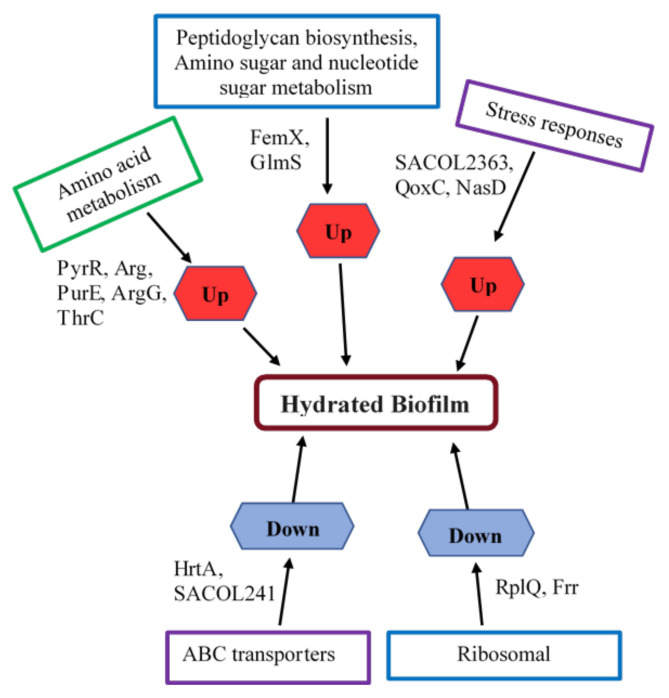
Potential regulators of *S. aureus* biofilm formation in the 12-day biofilm were identified from TMT-based analysis.

Of these proteins, glucosamine-fructose-6-phosphate aminotransferase (GlmS), and argininosuccinate synthase (ArgG) are cytoplasmic enzymes involved in alanine, aspartate and glutamate pathway. Galactose-6-phosphate isomerase (LacA) is a part of galactose metabolism pathway. These proteins are responsible for energy metabolism and are the vital points to support regular cell development and replication processes for biofilms [28,29,30]. In Figure 2, we have demonstrated potential regulators of *S. aureus* biofilm formation in the hydrated biofilm identified from TMT-based analysis.

Multiple studies reported the selective amino acid uptake for *S. aureus* biofilm mode of growth versus planktonic cultures [28,31,32,33] and suggested they may be an important feature distinguishing between planktonic and biofilm growth conditions. In our study, arginase encoded by Arg is significantly upregulated in hydrated biofilm and is involved in arginine metabolism, and is suggested to play a significant role in biofilm survival [31,32]. Ammons et al. (2014) and others have suggested that amino acid metabolism not only serves as a significant source of energy for biofilm growth but also initiates an adaptive approach to nutrient availability, redox balance, and environmental conditions [31,33].

A cytoplasmic enzyme aminoacyltransferase (FemX), involved in peptidoglycan biosynthesis pathway which is important for cell-wall synthesis, is upregulated. Other upregulated proteins include phosphoribosyl transferase (PyrR) involved in pyrimidine metabolism, and 5-(carboxyamino) imidazole ribonucleotide mutase (PurE) involved in both biosyntheses of secondary metabolites and purine metabolism. This demonstrates the use of secondary carbon sources for energy production, supporting the role of purine and pyrimidine metabolism in biofilm formation [28,33]. Other upregulated proteins are components in unrecognised pathways.

### 2.3. Sugar Transporter and Cell-Wall Synthesis Proteins Are Highly Abundant in the DSB

The proteins that are significantly upregulated in the DSB in comparison with hydrated biofilm are listed in Table 2. The full list of significantly upregulated proteins in the DSB is available in Table S4.

Of these, phosphotransferase system (PTS) glucose transporter subunit IIBC (PtaA) is significantly upregulated in DSB, suggesting that sugar uptake and PIA-mediated biofilm matrix deposition became active in DSB through PTS. Multiple proteomics analysis has demonstrated an increased abundance of PTS system-associated proteins under various biofilm growth stages and stress-associated conditions in several Gram-positive organisms including *S. aureus* [34,35,36]. Additionally, PtaA is also involved in amino sugar and nucleotide sugar metabolism and is responsible for energy metabolism and linked with peptidoglycan biosynthesis pathway. In particular, UDP-N-acetylmuramate-L-alanine ligase (MurC) and UDP-N-acetylenolpyruvoylglucosamine reductase (MurB) are cytoplasmic enzymes involved in the peptidoglycan biosynthesis pathway, which is essential for cell-wall formation and may play a role in biofilm formation. Cell-wall synthesis involves the construction of peptidoglycan monomers (polymeric mesh) from precursors UDP-N-acetylglucosamine and UDP-N-acetylmuramate [33].

**Figure 3:**
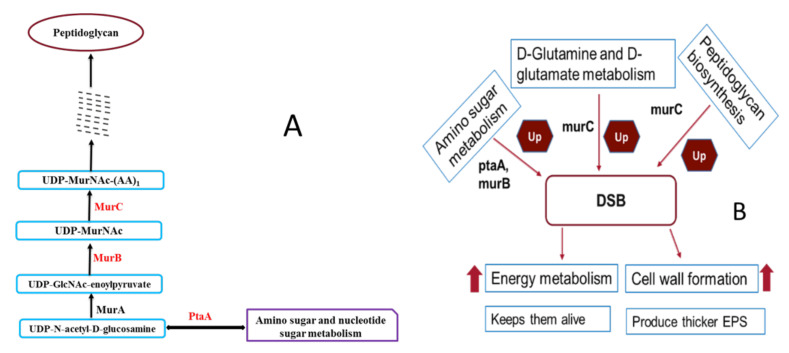
(**A**) Selected part of the peptidoglycan biosynthesis pathway and link with amino sugar metabolism; (**B**) Potential regulators of *S. aureus* biofilm formation in the DSB identified from TMT-based analysis.

They are synthesised in the cytoplasm of *S. aureus*, and simultaneously play a major role in maintaining structural and cellular integrity against osmotic forces within the environmental niche as well as permitting essential fluidity of the cell wall to adapt to changes in the cell shape during different stages of growth and division [37,38]. The metabolism associated with cell wall synthesis is well-recognised in *S. aureus*, and we observed a significantly higher abundance of precursor proteins in DSB which may contribute to the distinguishing features of the DSB in comparison with the hydrated biofilm and planktonic mode of growth. In addition, there was an increased abundance of amino and nucleotide sugar metabolism, and fermentation pathway-associated proteins, (such as PtaA, LacG, PurD), which are also involved in the production of cell wall components and EPS matrix deposition. Taken together, uniquely identified proteins associated with cell-wall synthesis and the link to energy metabolism associated proteins in DSB, keep cells alive and produce a thicker EPS matrix leading to increased resistance to biocides [10,12,18]. In Figure 3, we have demonstrated novel regulators of *S. aureus* biofilm formation in the DSB identified from TMT-based analysis.

In comparison between hydrated biofilm and DSB, we observed a relatively higher abundance of energy metabolism-associated proteins in the hydrated biofilm. Hydrated biofilm was constantly supplied with better nutrition (i.e., 20 versus 5% TSB) throughout its growth. In contrast, very strict, limited nutrients (5%) and water supply were provided to DSB during its growth. In this state, DSB are in an extreme nutrient-deficient condition in the deeper layer of cells [39]. In addition, a lower level of water content limits the penetration and consumption of nutrients into the adjacent cell clusters [40]. In this harsh condition, DSB utilizes major fermentation pathway-associated products because these products are situated just below the surface. In addition, we observed a higher abundance of cell-wall synthesis proteins in the DSB. The presence of a thicker cell wall would aid the biofilm to survive prolonged desiccation. Thicker cell walls are present in spores that are highly tolerant to desiccation [40].

### 2.4. Fatty Acid Pathway Could Be a Potential Target for DSB

**Figure 4:**
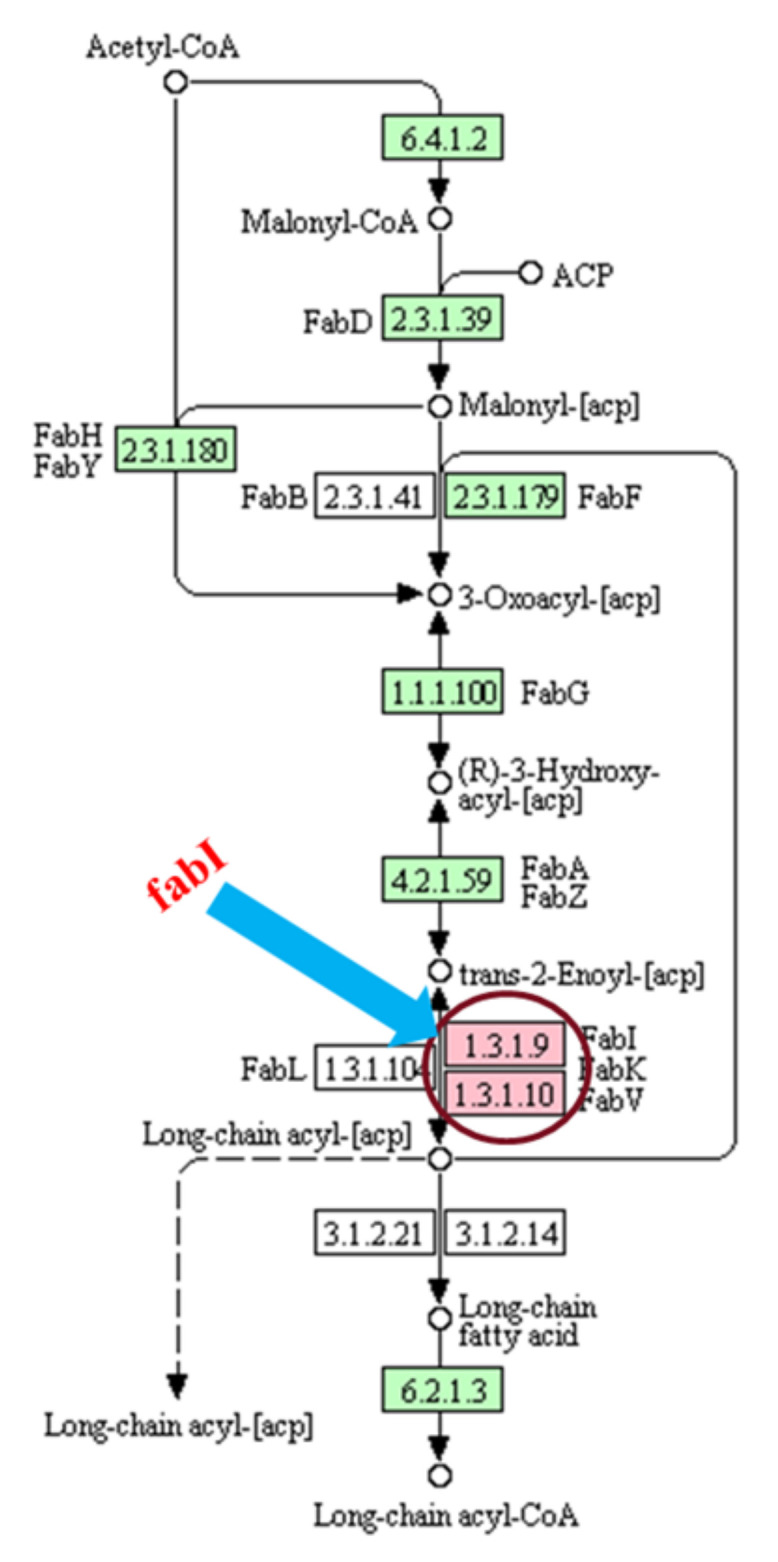
Selected part of type II fatty acid synthesis pathway for bacteria and the significant role of FabI in the last step of the elongation cycle in the synthesis of fatty acids.

A cytoplasmic membrane enzyme enoyl-ACP reductase (FabI) is a vital enzyme, involved in fatty acid biosynthesis and metabolism and was significantly upregulated in DSB. Enoyl-ACP reductase plays an essential role in the finishing cycles of the chain elongation process in the essential fatty acid biosynthesis pathway and is vital for cell survival (Figure 4) [41].

FabI is a remote member of an expanded protein family called short-chain alcohol reductases/dehydrogenases consisting of the motif Tyr-Xaa_6_-Lys. Multiple studies suggested that this enzyme might be a potential target for biocides to treat bacterial infections including biofilm-associated infections [41,42,43,44,45]. FabI is the target of the broad-spectrum biocides such as triclosan [46,47], and hexachlorophene [42,48]. Therefore, the significant upregulation of FabI enzyme we identified in this study may play a pivotal role in biofilm formation through the fatty acid synthesis pathway.

### 2.5. Ribosomal and ABC Transporter Proteins Revealed Decreased Abundance in the Hydrated Biofilm

The proteins that are significantly downregulated in the hydrated biofilm are listed in Table 3. In addition, the full list of significantly downregulated proteins in the hydrated biofilm is available in Table S5.

Of these, a cytoplasmic protein 50S ribosomal protein L17 (RplQ) is a part of the ribosomal pathway and changes in ribosomal protein expression can help cells adapt to diverse conditions and guarantee cellular metabolism. It is expected that cells make relative transcription and translation changes to create differential regulatory proteins to meet the necessities of various conditions. Hemin ABC transporter ATP-binding protein (HrtA) and amino acid ABC transporter substrate-binding protein (SACOL2412), are significantly downregulated in hydrated biofilm and are involved in ABC transporter systems. This suggests that the active transport of hemin and amino acid are downregulated in the hydrated biofilm, and several ABC transporter proteins have been demonstrated to play a role in *S. aureus* pathogenesis and antimicrobial resistance [49,50,51,52]. For example, *S. aureus* relies on various amino acids under conditions of limited resources and multiple amino acid transporters play an important role to fulfil their metabolic necessities for virulence, growth, and persistence [51]. Furthermore, studies have shown that ABC transporter of *S. aureus* reduces virulence by changing the structure and function of the cell wall [53] and, multiple omics studies have reported differential expression of ABC transporter proteins during biofilm growth [35,54,55,56,57]. Sulfite reductase [NADPH] flavoprotein alpha-component (SA2413), and cell division protein (FtsY) are cytoplasmic membrane proteins involved in different metabolic pathways such as microbial metabolism, sulfur metabolism, protein export and bacterial secretion system, respectively. Other downregulated proteins include GTP pyrophosphokinase (SA0864) associated with purine metabolism. Additional proteins such as hypothetical protein KQ76_13105 (SACOL2519), transcription elongation factor (GreA), acyl carrier protein (AcpP), etc. are cytoplasmic proteins involved in unrecognised pathways.

In contrast, the proteins that are significantly downregulated in the DSB are listed in Table S6. Of these, glycerophosphodiester phosphodiesterase (SACOL1770) is a part of glycerophospholipid metabolism. RNA polymerase encoded by SigB is a cytoplasmic enzyme belonging to sigma factor protein. Proteins involved in unrecognised pathways include membrane protein (cytoplasmic membrane) encoded by SACOL1113, ribonuclease III (MrnC), cell division protein ZapA, etc.

### 2.6. Significant Differentially Expressed Stress Response Proteins in the Hydrated Biofilm and DSB

In this novel study, we identified several unique/significant differentially expressed proteins associated with stress responses in hydrated biofilm such as L-lactate permease (SACOL2363), cytochrome C oxidase subunit III (QoxC), general stress protein (SA1692), thioredoxin (SA0758), glyoxal reductase (SA1606), and in DSB such as nitrite reductase (NasE), thiol peroxidase (SA1680), and pyridine nucleotide-disulfide oxidoreductase (SACOL1821). Among them, L-lactate permease regulates uptake and utilisation of L-lactate, and studies have reported that L-lactate uptake and utilisation are influenced by various stress response conditions such as oxygen limitation and acid stress [58,59,60], and play a significant role in biofilm formation under such diverse environments [61,62,63]. Acquisition of L-lactate has been shown to promote bacterial colonisation and enhance pathogenesis [61]. Under hypoxic conditions, a sudden drop in NAD^+^ regeneration and ATP synthesis increases glycolytic activity and activates fermentation pathways. In the hydrated biofilm, we observed significant upregulation of L-lactate permease suggesting it may have a pivotal role in biofilm formation or maintenance. The upregulation of L-lactate permease was also reported in a transcriptomics study by Morrison et al. (2018), where they grew *S. aureus* culture in the human spaceflight environment and their corresponding ground control cultures. In the same study, they observed L-lactate permease was downregulated in the *Bacillus subtilis* [64]. Another study by Clark et al. (2012), reported that L-lactate permease was downregulated in *Desulfovibrio vulgaris* biofilm growth mode compared to planktonic culture [63]. In addition, cytochrome C oxidase subunit III (QoxC) was significantly upregulated in the hydrated biofilm, and it is involved in electron transport chain maintenance under different stress conditions within the biofilm.

On the other hand, nitrite reductase (NasE) was significantly upregulated in the DSB and plays a major role in energy production under anaerobic/hypoxic conditions. Our study revealed a higher accumulation of cell-wall synthesis-associated proteins and Almatroudi et al. study [18] showed thicker EPS in the DSB which suggest that the synthesis of increased cell-wall components is a mechanism to ensure the maintenance of a critical level of hydration within the DSB. Hydrated biofilm is composed of approximately 90% water whilst DSB only has 61% water [39]. Variation in the water content limits the penetration of sufficient solute to the interior of cell clusters. Studies by Stewart (2003), demonstrated that limited water content affects the penetration of oxygen in an adjacent cell cluster due to consumption and diffusion impairment [40]. Therefore, deeper layers of biofilm are in a state of oxygen limitation; therefore, alternative substrates for oxidation are required to maintain cell aggregation and stabilisation. Fermentation products such as nitrite, which are present just below the biofilm surface, utilise the oxygen from the surface to maintain cell aggregation and biofilm stabilisation. A recent study by Graf et al. (2019) reported that, in an oxygen-limited environment, the strong positive charge created by fermentation products most likely mediates electrostatic interactions with anionic cell surface components, eDNA, and anionic metabolites leading to strong cell aggregation and biofilm stabilisation [29]. The deeper layers of cells are therefore subjected to growth-limiting circumstances, with anaerobic or micro anaerobic surroundings. Nitrite reductase could support these cells, confirming their survival without or with low oxygen. Oxygen-limitation has been reported for biofilms of several species including *S. aureus* [28,29,57,65,66,67]. In addition, the water availability in the DSB is very low compared to hydrated biofilm, suggesting DSB cells may also be supported by NasE under limited water content.

### 2.7. Scanning Electron Microscopy Observation of Hydrated Biofilm and DSB

**Figure 5:**
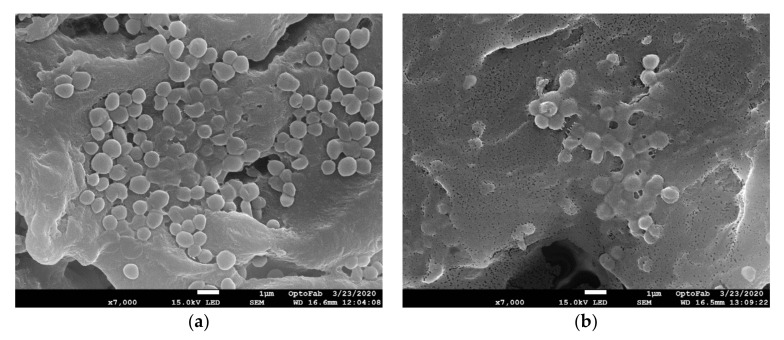
Scanning electronic micrographs of *S. aureus* biofilm (**a**) Hydrated biofilm (**b**) Dry Surface Biofilm (DSB).

To further extend our understanding we performed scanning electron microscopy (SEM) of hydrated biofilm and DSB (Figure 5). SEM analysis confirmed the formation of bacterial biofilms on polycarbonate coupons as well as there were distinguishable differences between the different biofilms in the amount of EPS present. In the hydrated biofilm, we observed many of the cells were embedded in the EPS, it was still easy to see individual cells sitting on top of the EPS surface or partly covered by the EPS (Figure 5a). On the other hand, the EPS matrix was densest in the DSB, and it was difficult to discern individual cocci (Figure 5b). This phenotypic observation strongly supports the proteomics findings regarding a higher abundance of cell wall-associated proteins leading to thicker EPS matrix deposition in the DSB compared with hydrated biofilm.

### 2.8. Validation of TMT Data with qPCR Results

The ratios from the qPCR results were obtained by comparing with planktonic in hydrated biofilm and DSB. Upregulated and downregulated protein expression and gene expression results were expressed in fold change (FC). qPCR results showed that, in DSB, *Pyc* was significantly downregulated in both qPCR and TMT analysis. In a hydrated biofilm, *prs* was significantly upregulated in both qPCR and TMT analysis. Between hydrated biofilm and DSB, *SspA* was significantly downregulated in both qPCR and TMT analysis, indicating a relative consistency for qPCR with the TMT data. However, there was no correlation between qPCR and TMT data for genes (*MurBC* and *SdhB*) (Table S7 in Supplementary Materials).

The qPCR analysis showed that the levels of gene expression identified were only partially in line with our mass-spec-based TMT quantitation analysis. The poor correlation identified between the results from transcriptomics and proteomics analysis could be due to disparities in the half-lives and post-transcription machinery of the samples as well as differences in sample preparation methods such as cell lysis and extraction.

In this study, we have produced hydrated biofilm and DSB by following the same procedure for proteomics and qPCR validation analysis. Due to the unavoidable standard sample processing differences, which can be reflected on the partial variation in the qPCR validation analysis. To minimise this limitation, we have performed phenotype analysis using SEM which strongly supports the significant differences in the EPS matrix deposition between hydrated biofilm and DSB. Future studies can be included targeted mutational, regulatory mechanisms, and advanced microscopic analysis using potential candidates identified in this study.

In this study, we specifically focused on only extractomes (extractable cellular proteins) to explore the proteome changes that exist between biofilms grown under different conditions. Identified potential marker proteins reported in this study might add valuable information to the global proteome repository, especially novel dry surface biofilm proteome. However, further deep fractionation studies of different strains of *S. aureus* might be helpful to explore the large-scale differences between hydrated biofilm and DSB. Additionally, another limitation of this study is that the proteomics profile described in this paper occurred with bacteria growing on an inert surface and the profile may significantly change depending on the type of dry surfaces the biofilms are growing on.

## 3. Materials and Methods

The objective of this research was to create quantitative proteomic profiling to define the distinctions between *S. aureus* cells that progress through the lifestyles of hydrated biofilm and DSB. To accomplish this, we conducted protein extraction and fractionation, reduction, alkylation and in-solution digestion that enabled us to produce samples that were analysed with TMT-based mass spectrometry. Three biological replicates were analysed for each growth condition: planktonic, prolonged biofilms and DSB.

### 3.1. Microorganism and Culture Conditions

*S. aureus* reference strain (ATCC 25923) was grown to stationary phase in 100% tryptic soy broth (TSB) for 24 h with constant agitation at 130 rpm and 37 °C. *S. aureus* 12-day biofilm was grown according to our previously published method [68]. Briefly, hydrated biofilm was grown in tryptic soy broth (TSB) on removable polycarbonate coupons in the CDC biofilm reactor (BioSurface Technologies Corp, Bozeman, MT, USA) over 12 days in batch phase (50% TSB for 48 h the media was then changed every 48 h with 20% TSB, at 37 °C with 130 rpm). *S. aureus* DSB was grown according to Almatroudi’s method [18]. Briefly, 1 mL of liquid culture of *S. aureus* (10^8^/mL) was added to the CDC biofilm reactor vessel containing 500 mL sterile 5% TSB and biofilm grown in batch phase, at 35 °C for 48 h followed by 48 h of dehydration at RT. Additionally, three more cycles of batch culture for 6 h at 35 °C, were interspersed with prolonged dehydration phases of 66, 42 and 66 h at RT [18]. Biofilm was grown and harvested from three separate experiments.

### 3.2. Protein Extraction and Fractionation

Protein extraction and fractionation were performed according to our previous published method [68]. Briefly, planktonic bacteria were pooled from three independent growth of 24 h *S. aureus* cultures and were mixed with lysis buffer containing 100 mM Triethylammonium bicarbonate (TEAB; Sigma-Aldrich, St. Louis, MO, USA) pH 8.5 and 1% (*w/v*) sodium deoxycholate (Sigma-Aldrich) at 10:1 ratio (supernatant: lysis buffer), while *S. aureus* biofilms (12-day biofilm and DSB) coated coupons (*n* = 24) were washed to remove non-adherent cells and coupons individually placed in 2 mL of phosphate-buffered saline (PBS) and lysis buffer and incubated overnight with gentle shaking at 4 °C. Samples were probe sonicated in an ice-cold environment (Sonic Ruptor, Omni International, Kennesaw, Georgia, GA, USA) for 2 min at 50% power and 70% pulses. The samples were centrifuged at 12,000× *g* for 10 min and the supernatant was filtered through a 10 kDa molecular weight cut-off (MWCO) ultra-membrane filter tube (Sigma-Aldrich) before centrifugation at 4000× *g* for 20 min. Protein samples were washed three times with PBS to remove TSB and lysis buffer and further concentrated using a 3 KDa MWCO filter tube (Sigma Aldrich).

The BCA protein assay (Thermo Fisher Scientific, Waltham, MA, USA) was used to measure protein concentration at 562 nm wavelength as per the manufacturer’s instructions.

### 3.3. Protein Reduction, Alkylation, and Digestion 

Protein reduction, alkylation, and digestion were performed according to our previous published method [68]. Briefly, a total of 40 µg protein of each biological replicate was reduced with 5 mM DTT for 15 min at RT and alkylated with 10 mM iodoacetamide (IAA) in the dark for 30 min at RT. Alkylated samples were diluted with 100 mM TEAB pH 8.5. Samples were digested overnight at RT by the addition of Lys-C at a ratio of 1:30 followed by the addition of trypsin at a ratio of 1:30 for 5.5 h at 37°C. Samples were adjusted to 1% (*v*/*v*) TFA, and the precipitated deoxycholate was removed by centrifugation. Samples were centrifuged at 14,100× *g* and desalted with 0.2% TFA washing using SDB-RPS (3M-Empore) Stage Tips (Thermo Fisher Scientific). Samples were eluted with 5% ammonium hydroxide in 80% Acetone and centrifuged at 1000× *g* for 5 min, vacuum dried and stored at −20 °C until further processing.

### 3.4. TMT Labelling and High pH Fractionation

TMT labelling and high pH fractionation were performed according to our previous published method [68]. Briefly, TMT (Thermo Fisher Scientific) reagents (0.8 mg) were dissolved in Acetone (85 µL) of which 41 µL was added to the reconstituted (100 µL of 100 mM TEAB pH 8.5) samples and incubated for 1 h at RT. A volume of 8 µL of 5% Hydroxylamine was added to each TMT labelled sample and incubated for 15 min at RT. A volume of 2 µL of each labelled sample was pooled and vacuum dried and reconstituted in 30 µL 0.1% formic acid (FA) (Merck, Kenilworth, NJ, USA) solution, centrifuged for 5 min at 14,000× *g* and analysed with mass spectrometer (for detailed information see nanoLC-ESI-MS/MS using Orbitrap Elite). 

Data searching was conducted using Proteome Discoverer 1.3 (for detailed information see data processing). Based on the applied normalization values from this search result an equal number of peptides were taken from each sample, pooled and vacuum dried (miVac). The dried labeled sample was resuspended in buffer A (5 mM ammonia, pH 10.5) and fractionated by high pH reverse phase-high pressure liquid chromatography (RP-HPLC; Agilent Technologies, Santa Clara, CA, USA). The dried labeled sample was resuspended in buffer A. After sample loading and washing with 97% buffer A for 10 min, the concentration of buffer B (5 mM ammonia solution with 90% Acetone, pH 10.5) was increased from 3% to 30% for 55 min; 70% for 10 min; 90% for 5 min at a flow rate of 300 µL/min. The eluent was collected every 2 min at the beginning until 16 min and every 1 min intervals for the remainder of the gradient. The fractionated sample was pooled into 19 fractions, and dried in miVac. Finally, each fraction was resuspended in 55 µL of 0.1% FA for mass spectrometer (MS) analysis.

### 3.5. Nanoflow LC-ESI-MS/MS

All samples were run on two sequential mass spectrometer systems Orbitrap Elite (Thermo Fisher Scientific) and Q Exactive (Thermo Fisher Scientific) according to our previous published method [68].

#### 3.5.1. Nanoflow LC-ESI-MS/MS Using Orbitrap Elite

An Orbitrap Elite (Thermo Fisher Scientific) mass spectrometer equipped with PicoView 550 Nanospray Source (New Objectives), an Eksigent ultra-pressure liquid chromatography (UPLC) system (AB SCIEX) consisting of an ekspert™ nanoLC 425 UPLC pump and ekspert™ nanoLC 400 (Thermo Fisher Scientific) autosampler was used for acquiring data. 20 µL of each fraction was loaded onto a self-packed 100 µm × 3.5 cm trap column with Halo^®^ 2.7 µm 160 Å ES-C18 (Advanced Materials Technology, Wilmington, DE, USA) and desalted with loading buffer [0.1% FA] at a flow rate of 4 µL/min for 10 min. Peptides were eluted onto a self-packed analytical column 100 µm × 30 cm with Halo^®^ 2.7 µm 160 Å ES-C18 (Advanced Materials Technology) with the linear gradients of mobile phase A (0.1% FA/5% DMSO) and mobile phase B (0.1% FA/5% DMSO) starting with B (1–10%) for 0.1 min, B (10–20%) for 52 min, B (20–32%) for 48 min followed by (32–43%) for 20 min with a flow rate of 450 nL/min across the gradient. The eluent from the trap was diluted with 100 nL/min of buffer A before reaching the analytical column. The peptides refocused and separated over the analytical column at 60 °C. Peptides were ionized by electrospray ionization, and data-dependent MS/MS acquisition carried out using an Orbitrap Elite (Thermo Fisher Scientific) consisting of 1 full MS1 (R = 120 K) scan acquisition from 380 to 1600 *m*/*z*, and 15 higher energy collisional dissociation (HCD) type MS2 scans (R = 30 K).

#### 3.5.2. Nanoflow LC-ESI-MS/MS Using Q Exactive

A Q Exactive (Thermo Fisher Scientific) Mass Spectrometer equipped with Nano spray Source and Easy nLC 1000 (Thermo Fisher Scientific) was used for acquiring data. 10 µL of each fraction was loaded onto a self-packed 100 µm × 3.5 cm reversed-phase peptide trap with Halo^®^ 2.7 µm 160 Å ES-C18 (Advanced Materials Technology) desalted with 20 µL of loading buffer (0.1% FA) and the peptide trap was then switched on-line with the analytical column a self-packed 75 µm × 3.5 cm Halo^®^ 2.7 µm 160 Å ES-C18 column. Peptides were eluted with the linear gradients of mobile phase A (0.1% FA) and buffer B [100%(*v*/*v*) Acetone, 0.1%(*v*/*v*) FA] starting with (1–30%) for 110 min, B (30–85%) for 2 min followed by 85% B for 8 min with a flow rate of 300 nL/min. Peptides were ionized by electrospray ionization and data-dependent MS/MS acquisition was carried out using a Q-Exactive consisting of 1 full MS1 (R = 70 K) scan acquisition from 350 to 1850 *m*/*z*, and 10 HCD type MS2 scans (R = 70 K).

### 3.6. Database Search, Statistical Analysis, and Bioinformatics

Database search, statistical analysis, and bioinformatics were performed as described in our previous publication [68]. Briefly, the raw data files were submitted to Proteome Discoverer (version 1.3, Thermo Fisher Scientific). The data were processed using Sequest and Mascot (Matrix Science, London, UK) against *S. aureus* reference strain (ATCC 25923) from Genbank CP009361 and CP009362. For protein identification, the following options were used: peptide mass tolerance = 10 ppm; MS/MS tolerance = 0.1 Da; enzyme = trypsin, missed cleavage = 1; fixed modification, carbamidomethyl (C), TMT10-plex (K) and TMT10-plex (N-term); variable modification, oxidation (M), Deamidated (N, Q) and Acetyl (N-Terminus). Quantification was performed based on the peak intensities of reporter ions in the MS/MS spectra. Below 1% false discovery rate was selected as the cut-off for peptide identification. Protein quantification was based on the total intensity of the assigned peptides. After the extraction of protein ratios with Proteome Discoverer, additional processing and statistical analyses were performed using the TMTPrePro R package. BLAST search was performed using highly annotated strains *S. aureus* N315 and *S. aureus* COL. Proteins were considered upregulated when the TMT ratio was above 1.5 and downregulated when the TMT ratio was lower than 0.66 in biofilm growth compared to planktonic growth with a significant *p*-value < 0.05. Significant differentially expressed proteins (>2-fold) were determined by using VENNY (v.2.1) (http://bioinfogp.cnb.csic.es/tools/venny/, accessed on 25 June 2018) and processed further to gain more functional insights. Metabolic pathways of identified proteins were analysed by using Kyoto Encyclopedia of Genes and Genomes (KEGG) mapper (https://www.genome.jp/kegg/tool/map_pathway2.html, accessed on 6 August 2018). Subcellular localisation of identified proteins was analysed by using PSORTb (version 3.0.2) (http://www.psort.org/psortb/index.html, accessed on 23 January 2018). The protein–protein interaction (PPI) network of significantly differentially expressed proteins was analysed by STRING software v.10.0 (http://string-db.org/, accessed on 25 June 2018).

### 3.7. Validation of TMT Data with qPCR Results

Validation of TMT data with qPCR results was performed as described in our previous publication [68]. Six genes encoding UDP-N-acetylmuramate-alanine ligase *MurC*, UDP-N-acetylenolpyruvoylglucosamine reductase *MurB*, Glutamyl endopeptidase *SspA*, Ribose-phosphate pyrophosphokinase *Prs*, Pyruvate carboxylase *Pyc*, and Succinate dehydrogenase *SdhB* were chosen as targets to analyse the levels of RNA expression to validate the expression changes in biofilms (hydrated biofilm and DSB). The 16S rRNA was used as an endogenous control to normalise the data, and the level of differential expression of the six genes between planktonic and biofilms was compared. 

RNA was extracted from *S. aureus* planktonic, hydrated biofilm, and DSB using an RNeasy Mini Kit (Qiagen, Hilden, Germany) with RNAlater to prevent degradation and RNase-free DNase treatment to remove genomic DNA. RNA concentration was determined by absorbance at 260 nm, and quality was assessed by absorbance ratio (A260/A280). A total of 200 ng of RNA was used for cDNA synthesis using the SuperScript™ IV VILO™ Master Mix (Thermo Fisher Scientific). Real-time (RT)-PCR primers used in this study (Table S8) were designed following *S. aureus* (ATCC 25923) genome sequence Genbank accession number CP009361 and CP009362.

Quantitative real-time PCR (qPCR) was conducted in an Applied Biosystems quantitative real-time PCR machine (ViiA™ 7 qPCR, ThermoFisher Scientific) in duplicate on two biological replicates. A volume of 25 μL of qPCR reaction mix containing 12.5 μL 2X PowerUp™ SYBR™ Green Master Mix (Thermo Fisher Scientific), 1 μL each of 10 μM reverse and forward primer for a final primer concentration of 400 nM, 8.5 μL of water and 2 μL of 1:5 diluted cDNA. Controls in each run included a no template control (NTC) for each primer set, which consisted of all PCR components except cDNA template which was replaced by nuclease-free water. A no reverse transcription control (no RT control) was also included in the initial experiments for each targeted gene. No RT controls consisted of the cDNA sample with no reverse transcriptase enzyme to determine if there was contaminating genomic DNA in the RNA. Cycling conditions for real-time PCR were set as an initial activation step of 95 °C for 10 min to activate the polymerase, followed by 40 cycles of denaturation at 95 °C for 15 s, annealing at 50 °C or 55 °C for 40 s and extension at 72 °C for 30 s, or annealing and extension at 60 °C for 1 min.

The expressed copy number of each target gene was normalised to 16s rRNA copy number within the same growth condition first, and the levels of candidate gene expression of planktonic and biofilms (hydrated biofilm and DSB) were compared to study relative gene expression using a previously described method [69]. The ratios from the qPCR results were obtained by comparing with planktonic in hydrated biofilm and DSB.

### 3.8. Scanning Electron Microscopy Observation of Biofilm

Biofilm-covered coupons were fixed in 3% glutaraldehyde, dehydrated through alcohol and hexamethyldisilazane (HMDS, Sigma-Aldrich) before sputter coating with 20 nm gold film as described previously [70] and examined in a scanning electron microscope (JEOL JSM 7100F FESEM, Tokyo, Japan).

## 4. Conclusions

This is the first report using high-throughput TMT-based MS determining proteins in *S. aureus* DSB. Our result showed significant abundance variation in hydrated biofilm and DSB. We observed protein (Arg) involved in arginine metabolism, which is suggested to play a pivotal role in biofilm survival in hydrated biofilm and protein (FabI) involved in fatty acid biosynthesis pathway, which is suggested to play a vital role in cell survival in DSB. Further pathway analysis revealed that energy metabolism-associated proteins (GlmS, ArgG, and LacA) were significantly upregulated in the hydrated biofilm, whereas cell-wall-synthesis-associated proteins (MurBC) were significantly upregulated in the DSB. In this study, we identified novel regulators of *S. aureus* biofilm formation in DSB. Identified novel regulators (PtaA, MurBC) play significant roles in cell survival, and thicker EPS matrix deposition might be one mechanism by which DSB increases its persistence in biocides. The current study will help design advanced, targeted disinfectants and detergents to remove biofilms from dry environments by further exploring key regulators identified in this study. 

## Figures and Tables

**Table 1:** Functional classification of the significantly upregulated proteins in hydrated biofilm in comparison to DSB (*p* < 0.05).

Accession ID	Protein Name	Fold Change	Protein Pathways
AIO20800.1	phosphoribosyl transferase, PyrR	2.5	Pyrimidine metabolism
AIO20657.1	cytochrome C oxidase subunit III, QoxC	2.3	Oxidative phosphorylation
AIO21790.1	glucosamine--fructose-6-phosphate aminotransferase, GlmS	2.1	Biosynthesis of antibiotics, Alanine, aspartate and glutamate metabolism, Amino sugar and nucleotide sugar metabolism
AIO21830.1	galactose-6-phosphate isomerase, LacA	2.1	Galactose metabolism
AIO22037.1	nitrite reductase, NasD	2.1	Microbial metabolism in diverse environments, Nitrogen metabolism
AIO21798.1	Arginase, Arg	2.01	Biosynthesis of secondary metabolites, Biosynthesis of antibiotics, Biosynthesis of amino acids, Arginine biosynthesis, Arginine and proline metabolism
AIO21892.1	Aminoacyltransferase, FemX	2.01	Peptidoglycan biosynthesis
OOC91313.1	5-(carboxyamino)imidazole ribonucleotide mutase, PurE	2.01	Biosynthesis of secondary metabolites, Biosynthesis of antibiotics, Purine metabolism
AIO20536.1	argininosuccinate synthase, ArgG	2.01	Biosynthesis of secondary metabolites, Biosynthesis of antibiotics, Biosynthesis of amino acids, Arginine biosynthesis, Alanine, aspartate and glutamate metabolism
AIO20949.1	threonine synthase, ThrC	2.01	Microbial metabolism in diverse environments, Glycine, serine and threonine metabolism, Vitamin B6 metabolism, Biosynthesis of amino acids

**Table 2:** Functional classification of the significantly upregulated proteins in DSB in comparison to hydrated biofilm (*p* < 0.05).

Accession ID	Protein Name	Fold Change	Protein Pathways
OOC91425.1	serine acetyltransferase, CysE	2.3	Microbial metabolism in diverse environments, Cysteine and methionine metabolism, Sulfur metabolism, Biosynthesis of amino acids, Carbon metabolism
AIO21414.1	PTS glucose transporter subunit IIBC, PtaA	2.3	Amino sugar and nucleotide sugar metabolism, Phosphotransferase system
AIO21824.1	6-phospho-beta-galactosidase, LacG	2.1	Galactose metabolism
AIO20705.1	inositol monophosphatase, SACOL1116	2.1	Streptomycin biosynthesis, Inositol phosphate metabolism
AIO21431.1	UDP-N-acetylmuramate--alanine ligase, MurC	2.1	D-Glutamine and D-glutamate metabolism, Peptidoglycan biosynthesis
AIO20408.1	UDP-N-acetylenolpyruvoylglucosamine reductase, MurB	2.1	Amino sugar and nucleotide sugar metabolism, Peptidoglycan biosynthesis
AIO22036.1	nitrite reductase, NasE	2.1	Microbial metabolism in diverse environments, Nitrogen metabolism
AIO20589.1	enoyl-ACP reductase, FabI	2.1	Biotin metabolism, Fatty acid biosynthesis and metabolism,
AIO20891.1	Recombinase, RecA	2.01	Homologous recombination
AIO20673.1	phosphoribosylamine-glycine ligase, PurD	2.01	Biosynthesis of secondary metabolites, Purine metabolism

**Table 3:** Functional classification of the significantly downregulated proteins in hydrated biofilm in comparison to DSB (*p* < 0.05).

Accession ID	Protein Name	Fold Change	Protein Pathways
AIO21853.1	50S ribosomal protein L17, RplQ	0.4	Ribosome
AIO21993.1	hemin ABC transporter ATP-binding protein, HrtA	0.4	ABC transporters
AIO22260.1	sulfite reductase [NADPH] flavoprotein alpha-component, SA2413	0.4	Microbial metabolism in diverse environments, Sulfur metabolism
AIO20838.1	cell division protein FtsY	0.4	Protein export, Bacterial secretion system, Quorum sensing
AIO20584.1	GTP pyrophosphokinase, SA0864	0.5	Purine metabolism
AIO22050.1	amino acid ABC transporter substrate-binding protein, SACOL2412	0.5	ABC transporters

## Data Availability

Data are available via ProteomeXchange [71] with identifier PXD033499.

## Supplementary Materials

The following supporting information can be downloaded at: https://www.mdpi.com/article/10.3390/ijms232012238/s1.
