# Supplementary material for: One Step Forward with Dry Surface Biofilm (DSB) of Staphylococcus aureus: TMT-Based Quantitative Proteomic Analysis Reveals Proteomic Shifts between DSB and Hydrated Biofilm
Source: Int J Mol Sci. 2022 Oct 13;23(20):12238. doi: 10.3390/ijms232012238 (PMC9602640; doi:10.3390/ijms232012238)
Supplement: Supplementary file 1 [file ijms-23-12238-s001.zip › ijms-1843530-supplementary.pdf]

**Table S1:** Functional classification of the significantly upregulated common proteins between hydrated biofilm and DSB compared with planktonic culture (p <0.05). *FC denotes fold change; ** CM denotes Cytoplasmic Membrane.

| Accession ID | Protein Name | Gene Name | Hydrated biofilm (FC)* | DSB (FC)* | Protein Pathways | Subcellular Localization |
|---|---|---|---|---|---|---|
| AIO22223.1 | type II secretion protein | SA2371 | 3.59 | 2.46 | | Unknown |
| AIO21382.1 | isocitrate dehydrogenase | icd citC SA1517 | 3.06 | 2.17 | Metabolic pathways, Biosynthesis of secondary metabolites, Biosynthesis of amino acids, Biosynthesis of antibiotics, Microbial metabolism in diverse environments, Carbon metabolism, 2-Oxocarboxylic acid metabolism, Glutathione metabolism, Citrate cycle (TCA cycle) | Cytoplasmic |
| AIO20590.1 | membrane protein | SA0870 | 2.62 | 2.04 | | CM** |
| AIO22305.1 | hypothetical protein KQ76_13935 | SA2451 | 5.52 | 4.40 | | Unknown |
| OOC90965.1 | glutamine ABC transporter ATP-binding protein | SA1674 | 2.83 | 2.30 | | CM |
| AIO20668.1 | phosphoribosylformylglycin amidine synthase | purL SACOL1078 | 2.45 | 2.03 | Metabolic pathways, Biosynthesis of secondary metabolites, Purine metabolism, Biosynthesis of antibiotics | Cytoplasmic |
| AIO20812.1 | DNA-directed RNA polymerase subunit omega | rpoZ SA1053 | 3.74 | 3.16 | Metabolic pathways, Pyrimidine metabolism, Purine metabolism, RNA polymerase, | Cytoplasmic |

| | | | | | | |
|---|---|---|---|---|---|---|
| AIO20535.1 | argininosuccinate lyase | argH SA0821 | 2.43 | 2.06 | Metabolic pathways, Biosynthesis of secondary metabolites, Biosynthesis of amino acids, Biosynthesis of antibiotics, Alanine, aspartate and glutamate metabolism, Arginine biosynthesis | Cytoplasmic |
| AIO20780.1 | cell division protein MraZ | mraZ SA1021 | 2.58 | 2.21 | | Cytoplasmic |
| AIO19852.1 | branched-chain amino acid transporter II carrier protein | SA0180 | 3.81 | 3.27 | | CM |
| AIO20178.1 | dihydropteroate synthase | folP SA0472 | 3.28 | 2.81 | Metabolic pathways, Folate biosynthesis | CM |
| AIO20178.1 | dihydropteroate synthase | folP SA0472 | 3.28 | 2.81 | Metabolic pathways, Folate biosynthesis | Cytoplasmic |
| AIO21773.1 | membrane protein | SA1937 | 3.20 | 2.82 | | CM |
| AIO21226.1 | octanoyltransferase | lipM SA1363 | 2.68 | 2.38 | Metabolic pathways, Lipoic acid metabolism | Cytoplasmic |
| AIO20979.1 | sodium:alanine symporter | alsT | 2.67 | 2.42 | | CM |
| AIO20958.1 | guanosine 5'-monophosphate oxidoreductase | guaC SACOL1371 | 3.28 | 2.97 | Purine metabolism | Cytoplasmic |
| AIO21819.1 | hypothetical protein KQ76_11385 | SA1986 | 3.27 | 2.96 | | CM |
| AIO22040.1 | formate/nitrite transporter | SACOL2401 | 2.56 | 2.33 | | CM |
| AIO21727.1 | membrane protein | yidC SA1893 | 2.46 | 2.27 | Protein export, Bacterial secretion system, Quorum sensing | CM |
| AIO21864.1 | 50S ribosomal protein L18 | rplR SA2032 | 2.33 | 2.19 | Ribosome | Cytoplasmic |

| | | | | | | |
|---|---|---|---|---|---|---|
| AIO19795.1 | acetoin reductase | butA SA0122 | 2.49 | 2.35 | Butanoate metabolism | Cytoplasmic |
| AIO22226.1 | fructosamine kinase | SA2374 | 2.71 | 2.55 | | Cytoplasmic |
| AIO21765.1 | CTP synthetase | pyrG ctrA SA1929 | 2.32 | 2.24 | Metabolic pathways, Pyrimidine metabolism | Cytoplasmic |
| AIO21934.1 | octopine dehydrogenase | SA2095 | 2.20 | 2.13 | | Cytoplasmic |
| AIO20806.1 | orotidine 5'-phosphate decarboxylase | pyrF SA1047 | 2.54 | 2.49 | Metabolic pathways, Pyrimidine metabolism | Cytoplasmic |
| AIO21333.1 | ATP-dependent DNA helicase RuvB | ruvB SACOL169 6 | 2.55 | 2.50 | Homologous recombination | Cytoplasmic 9.98 |
| AIO21826.1 | PTS system lactose-specific transporter subunit IIA | lacF SA1993 | 2.36 | 2.32 | Metabolic pathways, Galactose metabolism, Phosphotransferase system (PTS) | Cytoplasmic 10.01 |

| | | | | | | |
|---|---|---|---|---|---|---|
| AIO20163.1 | ribose-phosphate pyrophosphokinase | prs SA0458 | 2.81 | 2.79 | Metabolic pathways, Biosynthesis of secondary metabolites, Biosynthesis of amino acids, Biosynthesis of antibiotics, Purine metabolism, Microbial metabolism in diverse environments, Carbon metabolism, Pentose phosphate pathway | Cytoplasmic |
| AIO21316.1 | hypothetical protein KQ76_08470 | SAS049 | 2.21 | 2.21 | | Unknown |
| AIO21860.1 | preprotein translocase subunit SecY | secY SA2028 | 2.15 | 2.16 | Protein export, Bacterial secretion system, Quorum sensing | CM |
| AIO21768.1 | pantothenate kinase | coaW SA1932 | 2.01 | 2.03 | Metabolic pathways, Pantothenate and CoA biosynthesis | Unknown |
| AIO20718.1 | glycerophosphodiester phosphodiesterase | SA0969 | 2.38 | 2.45 | Glycerophospholipid metabolism | Cytoplasmic |
| AIO21539.1 | pseudouridine synthase | SA1668 | 2.50 | 2.58 | | Cytoplasmic |
| AIO20864.1 | uridylate kinase | pyrH smbA SA1101 | 2.07 | 2.20 | Metabolic pathways, Pyrimidine metabolism | Cytoplasmic |
| AIO20697.1 | spermidine/putrescine ABC transporter ATP-binding protein | potA SA0950 | 2.30 | 2.46 | ABC transporters | CM |
| AIO21307.1 | hypothetical protein KQ76_08425 | SA1443 | 2.10 | 2.26 | | Cytoplasmic |
| AIO21769.1 | hypothetical protein KQ76_11075 | SA1933 | 2.10 | 2.26 | | Cytoplasmic |

| | | | | | | |
|---|---|---|---|---|---|---|
| AIO20807.1 | orotate phosphoribosyltransferase | pyrE SA1048 | 2.13 | 2.30 | Metabolic pathways, Pyrimidine metabolism | Cytoplasmic |
| AIO21210.1 | 2-oxoisovalerate dehydrogenase | SACOL156 1 | 3.11 | 3.36 | Metabolic pathways, Biosynthesis of secondary metabolites, Biosynthesis of antibiotics, Propanoate metabolism, Valine, leucine and isoleucine degradation | Cytoplasmic |
| AIO21335.1 | ACT domain-containing protein | SA1469 | 3.27 | 3.59 | Metabolic pathways, Biosynthesis of secondary metabolites, Biosynthesis of amino acids, Biosynthesis of antibiotics, Phenylalanine, tyrosine and tryptophan biosynthesis | Cytoplasmic |
| AIO22278.1 | amino acid APC transporter | arcD | 2.23 | 2.47 | | CM |
| AIO20787.1 | cell division protein FtsA | ftsA SA1028 | 2.94 | 3.29 | | Cytoplasmic |
| AIO21825.1 | PTS system lactose-specific transporter subunits IICB | lacE SACOL218 1 | 2.27 | 2.54 | Metabolic pathways, Phosphotransferase system (PTS), Galactose metabolism | CM |
| AIO21878.1 | 50S ribosomal protein L23 | rplW SA2045 | 2.15 | 2.41 | Ribosome | Cytoplasmic |
| AIO20778.1 | hypothetical protein KQ76_05635 | SA1019 | 2.41 | 2.71 | | Unknown |
| AIO21910.1 | molybdenum ABC transporter permease | modB | 2.57 | 2.93 | ABC transporters | CM |
| AIO21879.1 | 50S ribosomal protein L4 | rplD SA2046 | 2.03 | 2.33 | Ribosome | Unknown |
| AIO20968.1 | exonuclease | sbcD SA1180 | 2.36 | 2.73 | | Cytoplasmic |

| | | | | | | |
|---|---|---|---|---|---|---|
| AIO22176.1 | PTS system glucose-specific transporter subunit IICBA | glcB SACOL2552 | 2.07 | 2.40 | Phosphotransferase system (PTS), Amino sugar and nucleotide sugar metabolism, Glycolysis / Gluconeogenesis | CM |
| AIO22234.1 | pantoate--beta-alanine ligase | panC SACOL2614 | 2.91 | 3.42 | Metabolic pathways, Biosynthesis of secondary metabolites, Pantothenate and CoA biosynthesis, beta-Alanine metabolism | Cytoplasmic |
| AIO21337.1 | 50S ribosomal protein L27 | rpmA SA1471 | 2.64 | 3.12 | Ribosome | Cytoplasmic |
| AIO20205.1 | 50S ribosomal protein L10 | rplJ SA0497 | 2.16 | 2.69 | Ribosome | Cytoplasmic |
| AIO20817.1 | peptide deformylase | SA1058 | 2.10 | 2.66 | | Cytoplasmic |
| AIO19749.1 | hypothetical protein KQ76_00135 | | 2.73 | 3.48 | | Cytoplasmic |
| AIO20985.1 | LytR family transcriptional regulator | msrR SA1195 | 2.13 | 2.75 | | CM |
| AIO21707.1 | 4'-phosphopantetheinyl transferase | acpS dpj SA1875 | 2.37 | 3.18 | Pantothenate and CoA biosynthesis | |
| OOC94735.1 | hypothetical protein BWO94_01050 | | 3.63 | 4.91 | | Unknown |
| AIO20937.1 | hypothetical protein KQ76_06475 | SAS040 | 2.84 | 3.94 | | Unknown |
| AIO20620.1 | hypothetical protein KQ76_04815 | SACOL1585 | 2.20 | 3.15 | | Unknown |
| AIO20395.1 | lipid kinase | SACOL0787 | 2.05 | 3.03 | | Unknown |
| AIO21848.1 | 50S ribosomal protein L13 | rplM SA2017 | 2.14 | 3.28 | Ribosome | Unknown |

**Table S2:** Functional classification of the significantly downregulated common proteins between hydrated biofilm and DSB compared with planktonic culture ($p < 0.05$). *FC denotes fold change; ** CM denotes Cytoplasmic Membrane.

| Accession ID | Protein Name | Gene Name | Hydrated biofilm (FC)* | DSB (FC)* | Protein Pathways | Subcellular Localization |
|---|---|---|---|---|---|---|
| AIO20700.1 | spermidine/putrescine ABC transporter substrate-binding protein | potD | 0.08 | 0.04 | ABC transporters | Unknown |
| AIO22060.1 | gamma-hemolysin subunit A | hlgC SA2208 | 0.35 | 0.22 | *Staphylococcus aureus* infection | Extracellular |
| AIO20660.1 | chitinase | SA0914 | 0.09 | 0.06 | | Unknown |
| AIO20463.1 | thermonuclease | nuc SACOL0860 | 0.16 | 0.12 | | Extracellular |
| AIO21509.1 | serine protease | splC SACOL1867 | 0.28 | 0.20 | | Extracellular |
| AIO20279.1 | hypothetical protein KQ76_03005 | SA0570 | 0.48 | 0.36 | | Unknown |
| AIO19987.1 | lipase | lip2 geh SA0309 | 0.14 | 0.10 | Metabolic pathways, Glycerolipid metabolism | Extracellular |
| AIO20583.1 | hypothetical protein KQ76_04610 | SACOL1009 | 0.46 | 0.35 | | CM** |
| AIO20651.1 | mannosyl-glycoprotein endo-beta-N-acetylglucosamidase | atl nag SACOL1062 | 0.24 | 0.18 | | Extracellular |
| AIO21353.1 | delta-aminolevulinic acid dehydratase | hemB SA1492 | 0.27 | 0.21 | Metabolic pathways, Microbial metabolism in diverse environments, Biosynthesis of secondary metabolites, Porphyrin and chlorophyll metabolism | Cytoplasmic |

| | | | | | | |
|---|---|---|---|---|---|---|
| AIO20960.1 | secretion protein | SACOL1373 | 0.47 | 0.37 | | Unknown |
| AIO21128.1 | hypothetical protein KQ76_07495 | | 0.35 | 0.28 | | Cytoplasmic |
| OOC94758.1 | alpha/beta hydrolase | SA1990 | 0.33 | 0.26 | | Unknown |
| AIO20644.1 | cysteine protease | sspB SACOL1056 | 0.11 | 0.09 | | Extracellular |
| AIO19870.1 | peptidase M23 | SA0205 | 0.19 | 0.16 | | Extracellular |
| AIO20093.1 | superantigen-like protein | set11 | 0.33 | 0.28 | *Staphylococcus aureus* infection | Extracellular |
| AIO20534.1 | glycerophosphodiester phosphodiesterase | glpQ | 0.22 | 0.18 | | Unknown |
| AIO20738.1 | phenylalanyl-tRNA synthase subunit beta | pheT SA0986 | 0.38 | 0.33 | Aminoacyl-tRNA biosynthesis | Cytoplasmic |
| AIO22292.1 | adhesin | sasF SACOL2668 | 0.43 | 0.37 | | Cellwall |
| AIO21549.1 | Fur family transcriptional regulator | perR SACOL1919 | 0.48 | 0.42 | | Cytoplasmic |
| AIO21667.1 | delta-hemolysin | hld SA1841.1 SAS065 | 0.03 | 0.03 | Quorum sensing | Unknown |
| AIO20100.1 | hypothetical protein KQ76_01960 | SA0395 | 0.14 | 0.12 | | Unknown |
| AIO20951.1 | HAD family hydrolase | SA1167 | 0.43 | 0.37 | | Cytoplasmic |
| AIO20623.1 | conjugal transfer protein | SACOL1582 | 0.42 | 0.37 | | Unknown |
| AIO22349.1 | hypothetical protein KQ76_14165 | | 0.30 | 0.27 | | Unknown |
| AIO20056.1 | hypothetical protein KQ76_01725 | | 0.10 | 0.09 | | CM |

| | | | | | | |
|---|---|---|---|---|---|---|
| AIO21298.1 | acetyl-CoA carboxylase biotin carboxylase subunit | SA1434 | 0.41 | 0.36 | Metabolic pathways, Microbial metabolism in diverse environments, Biosynthesis of secondary metabolites, Biosynthesis of antibiotics, Carbon metabolism, Fatty acid metabolism, Propanoate metabolism, Pyruvate metabolism, Fatty acid biosynthesis | Cytoplasmic |
| AIO19780.1 | MarR family transcriptional regulator | sarS sarH1 SA0108 | 0.45 | 0.40 | | Cytoplasmic |
| AIO20712.1 | pyruvate carboxylase | pyc SACOL1123 | 0.50 | 0.45 | Metabolic pathways, Pyruvate metabolism, Microbial metabolism in diverse environments, Biosynthesis of amino acids, Citrate cycle (TCA cycle), Carbon metabolism | Cytoplasmic |
| AIO19770.1 | 1-phosphatidylinositol phosphodiesterase | plc | 0.19 | 0.17 | Inositol phosphate metabolism | Extracellular |
| AIO22290.1 | N-acetylmuramoyl-L-alanine amidase | SACOL2666 | 0.14 | 0.12 | | Extracellular |
| AIO20332.1 | peptidase M23B | SA0620 | 0.14 | 0.13 | | Cell wall |
| AIO20756.1 | formyl peptide receptor-like 1 inhibitory protein | flr SA1001 | 0.35 | 0.32 | *Staphylococcus aureus* infection | Unknown |
| AIO21349.1 | DNA-3-methyladenine glycosylase | tag | 0.40 | 0.36 | Base excision repair | Unknown |
| AIO21997.1 | LytTR family transcriptional regulator | SA2153 | 0.43 | 0.40 | | Unknown |

| | | | | | | |
|---|---|---|---|---|---|---|
| AIO21607.1 | nitric oxide synthase | nos SA1730 | 0.44 | 0.40 | Metabolic pathways, Biosynthesis of secondary metabolites, Arginine biosynthesis, Arginine and proline metabolism | Cytoplasmic |
| AIO20419.1 | DegV domain-containing protein | SACOL0812 | 0.49 | 0.45 | | Cytoplasmic |
| AIO21983.1 | esterase | SA2140 | 0.48 | 0.44 | | Cytoplasmic |
| AIO22180.1 | LysR family transcriptional regulator | SA2330 | 0.34 | 0.32 | | Cytoplasmic |
| AIO20126.1 | N-acetylmuramoyl-L-alanine amidase | sle1 aaa SA0423 | 0.29 | 0.27 | | Cellwall |
| AIO21449.1 | leucyl-tRNA synthetase | leuS SA1579 | 0.36 | 0.34 | Aminoacyl-tRNA biosynthesis | Cytoplasmic |
| AIO20739.1 | ribonuclease HIII | rnhC SACOL1150 | 0.22 | 0.20 | DNA replication | Unknown |
| AIO20704.1 | hypothetical protein KQ76_05255 | SA0957 | 0.49 | 0.46 | | Cytoplasmic |
| AIO20387.1 | glycerol phosphate lipoteichoic acid synthase | ltaS SA0674 | 0.15 | 0.14 | Metabolic pathways, Glycerolipid metabolism | CM |
| OOC91307.1 | hypothetical protein BWO94_08325 | SA1008 | 0.50 | 0.47 | | Unknown |
| AIO21583.1 | ferritin | ftnA SA1709 | 0.44 | 0.42 | | Cytoplasmic |
| AIO21618.1 | hypothetical protein KQ76_10250 | SAS056 | 0.46 | 0.44 | | |
| AIO19947.1 | staphyloxanthin biosynthesis protein | SACOL0270 | 0.13 | 0.12 | | Extracellular |
| AIO21246.1 | penicillin-binding protein | pbp3 | 0.36 | 0.35 | Metabolic pathways, beta-Lactam resistance, Peptidoglycan biosynthesis | CM |
| AIO20202.1 | antitermination protein NusG | nusG SA0494 | 0.46 | 0.44 | | Cytoplasmic |

| | | | | | | |
|---|---|---|---|---|---|---|
| OOC94232.1 | aureolysin | aur SACOL2659 | 0.32 | 0.30 | *Staphylococcus aureus* infection, Cationic antimicrobial peptide (CAMP) resistance | Extracellular |
| AIO19779.1 | peptigoglycan-binding protein LysM | spa SACOL0095 | 0.16 | 0.15 | *Staphylococcus aureus* infection | Cellwall |
| AIO20863.1 | elongation factor Ts | tsf SACOL1276 | 0.36 | 0.35 | | Cytoplasmic |
| AIO22317.1 | lipase | lip1 SA2463 | 0.20 | 0.19 | Metabolic pathways, Glycerolipid metabolism | Extracellular |
| AIO22350.1 | peptidase | | 0.13 | 0.12 | | Extracellular |
| AIO20885.1 | zinc protease | SACOL1298 | 0.46 | 0.44 | | Unknown |
| AIO20273.1 | arginine-tRNA ligase | argS SACOL0663 | 0.48 | 0.47 | Aminoacyl-tRNA biosynthesis | Cytoplasmic |
| AIO21182.1 | transcriptional regulator | srrA SA1323 | 0.33 | 0.32 | Two-component system | Cytoplasmic |
| AIO20196.1 | cysteinyl-tRNA synthetase | cysS SACOL0576 | 0.41 | 0.40 | Aminoacyl-tRNA biosynthesis | Cytoplasmic |
| AIO20645.1 | glutamyl endopeptidase | sspA SACOL1057 | 0.15 | 0.15 | Quorum sensing | Extracellular |
| AIO20455.1 | hypothetical protein KQ76_03935 | SACOL0851 | 0.42 | 0.41 | | Unknown |
| AIO22160.1 | lactate dehydrogenase | ldhD ddh SACOL2535 | 0.29 | 0.29 | Pyruvate metabolism, Microbial metabolism in diverse environments | Cytoplasmic |
| AIO21406.1 | Free methionine-(R)-sulfoxide reductase | SACOL1768 | 0.26 | 0.25 | Cysteine and methionine metabolism | Cytoplasmic |
| AIO22099.1 | peptidase M28 | SA2244 | 0.32 | 0.31 | | Cytoplasmic |
| AIO20194.1 | glutamyl-tRNA synthetase | gltX SA0486 | 0.39 | 0.39 | Metabolic pathways, Aminoacyl-tRNA biosynthesis | Cytoplasmic |
| AIO22275.1 | clumping factor B | clfB SA2423 | 0.33 | 0.33 | *Staphylococcus aureus* infection | Cellwall |
| AIO21805.1 | uridylyltransferase | SA1974 | 0.36 | 0.36 | | Cytoplasmic |

| | | | | | | |
|---|---|---|---|---|---|---|
| OOC93971.1 | phosphoglucomutase | pgcA SA2279 | 0.41 | 0.41 | Metabolic pathways, Microbial metabolism in diverse environments, Biosynthesis of secondary metabolites, Biosynthesis of antibiotics, Purine metabolism, Galactose metabolism, Glycolysis / Gluconeogenesis, Glycerophospholipid metabolism, Pentose phosphate pathway, Amino sugar and nucleotide sugar metabolism, Streptomycin biosynthesis, Starch and sucrose metabolism | Cytoplasmic |
| OOC94101.1 | DUF2648 domain-containing protein | SACOL2649 | 0.50 | 0.51 | | Unknown |
| AIO19942.1 | peptidase M23 | lytM SACOL0263 | 0.12 | 0.12 | | Extracellular |
| AIO21492.1 | hypothetical protein KQ76_09365 | SACOL1852 | 0.16 | 0.16 | | Unknown |
| AIO19900.1 | nitric oxide dioxygenase | SA0231 | 0.34 | 0.35 | | Cytoplasmic |
| AIO21508.1 | serine protease | splE SACOL1865 | 0.40 | 0.41 | | Extracellular |
| AIO20228.1 | hydrolase | sdrD SA0520 | 0.22 | 0.22 | *Staphylococcus aureus* infection | Cellwall |
| AIO20556.1 | hypothetical protein KQ76_04470 | SA0841 | 0.08 | 0.08 | | CM |
| AIO22338.1 | adhesin | | 0.46 | 0.47 | | Cellwall |
| OOC90902.1 | calcium-binding protein | SACOL1846 | 0.05 | 0.05 | | Unknown |
| AAX11326.1 | enterotoxin type Gv precursor | entG seg SA1642 | 0.40 | 0.41 | *Staphylococcus aureus* infection | Extracellular |
| AIO22285.1 | hypothetical protein KQ76_13835 | SACOL2661 | 0.37 | 0.39 | | Extracellular |

| | | | | | | |
|---|---|---|---|---|---|---|
| AIO20759.1 | fibrinogen-binding protein | SA1004 | 0.47 | 0.50 | *Staphylococcus aureus* infection | Unknown |
| AIO22081.1 | sodium:proton antiporter | SA2228 | 0.21 | 0.22 | | CM |
| AIO22369.1 | phosphodiesterase | hlb SACOL2003 | 0.12 | 0.12 | Metabolic pathways, Quorum sensing, Biosynthesis of secondary metabolites | Extracellular |
| AIO22017.1 | hypothetical protein KQ76_12415 | SA2168 | 0.39 | 0.42 | | Unknown |
| AIO21033.1 | hypothetical protein KQ76_06965 | SA1242 | 0.46 | 0.49 | | Unknown |
| AIO20783.1 | penicillin-binding protein | pbpA | 0.39 | 0.42 | Metabolic pathways, beta-Lactam resistance, Peptidoglycan biosynthesis | CM |
| AIO21282.1 | competence protein ComE | comEB | 0.41 | 0.44 | Metabolic pathways, Pyrimidine metabolism | Cytoplasmic |
| AIO20229.1 | bis(5'-nucleosyl)-tetraphosphatase | sdrE SACOL0610 | 0.26 | 0.28 | *Staphylococcus aureus* infection | Cellwall |
| AIO21274.1 | molecular chaperone DnaK | dnaK SA1409 | 0.43 | 0.47 | RNA degradation | Cytoplasmic |
| AIO22174.1 | thioredoxin | SA2324 | 0.42 | 0.47 | | Cytoplasmic |
| AIO20145.1 | DNA polymerase III subunit delta' | SACOL0526 | 0.31 | 0.34 | Metabolic pathways, DNA replication, Pyrimidine metabolism, Purine metabolism, Homologous recombination, Mismatch repair | Cytoplasmic |
| AIO20763.1 | alpha-hemolysin | SA1007 | 0.06 | 0.06 | | Extracellular |
| AIO20737.1 | phenylalanine--tRNA ligase | pheS SA0985 | 0.35 | 0.39 | Aminoacyl-tRNA biosynthesis | Cytoplasmic |
| AIO21788.1 | HAD family hydrolase | SA1957 | 0.30 | 0.34 | | Cytoplasmic |
| AIO20874.1 | 50S ribosomal protein L7 | SA1111 | 0.34 | 0.38 | | Unknown |

| | | | | | | |
|---|---|---|---|---|---|---|
| AIO21867.1 | 30S ribosomal protein S14 | rpsZ rpsN1 SA2034.1 SAS079 | 0.26 | 0.29 | Ribosome | Cytoplasmic |
| AIO20189.1 | excinuclease ABC subunit B | SA0481 | 0.36 | 0.40 | | Cytoplasmic |
| OOC92415.1 | sphingomyelin phosphodiesterase | hlb SACOL2003 | 0.33 | 0.37 | Glycerophospholipid metabolism, Inositol phosphate metabolism | Extracellular |
| AIO21275.1 | heat shock protein GrpE | grpE SA1410 | 0.36 | 0.41 | | Cytoplasmic |
| AIO20225.1 | HAD family hydrolase | SA0517 | 0.42 | 0.48 | | Cytoplasmic |
| AIO20637.1 | acetyltransferase | SA0893 | 0.17 | 0.20 | | Cytoplasmic |
| AIO22130.1 | DNA mismatch repair protein MutT | SACOL2500 | 0.41 | 0.47 | | Unknown |
| AIO20418.1 | ABC transporter | SACOL0811 | 0.27 | 0.31 | | Cytoplasmic |
| AIO21842.1 | toxin | SACOL2197 | 0.40 | 0.46 | | CM |
| AIO22387.1 | replication-associated protein (plasmid) | SAP031 | 0.41 | 0.48 | | Unknown |
| AIO20884.1 | zinc protease | SA1121 | 0.29 | 0.34 | | Cytoplasmic |
| AIO22136.1 | fibronectin-binding protein | fnbA SA2291 | 0.23 | 0.26 | Bacterial invasion of epithelial cells | Cellwall |
| AIO20222.1 | deoxycytidine kinase | SA0514 | 0.43 | 0.50 | Metabolic pathways, Purine metabolism, Pyrimidine metabolism | Cytoplasmic |
| AIO21273.1 | molecular chaperone DnaJ | dnaJ SA1408 | 0.21 | 0.24 | | Cytoplasmic |
| AIO22207.1 | transglycosylase | isaA SACOL2584 | 0.11 | 0.14 | | Extracellular |

| | | | | | | |
|---|---|---|---|---|---|---|
| AIO19907.1 | PTS sugar transporter subunit IIA | SA0236 | 0.24 | 0.29 | Metabolic pathways, Galactose metabolism, Phosphotransferase system (PTS) | Cytoplasmic |
| AIO20259.1 | dihydrolipoamide dehydrogenase | SA0551 | 0.42 | 0.51 | | Cytoplasmic |
| AIO21343.1 | membrane protein | SA1476 | 0.30 | 0.37 | | Unknown |
| AIO20071.1 | NADPH-dependent oxidoreductase | nfrA SACOL0453 | 0.26 | 0.32 | Metabolic pathways, Riboflavin metabolism | Cytoplasmic |
| AIO21601.1 | cysteine protease | sspP scpA SA1725 | 0.23 | 0.29 | | Extracellular |
| AIO20406.1 | iron ABC transporter substrate-binding protein | SA0691 | 0.35 | 0.44 | ABC transporters | CM |
| AIO20716.1 | SCP-like extracellular protein | SA0967 | 0.17 | 0.22 | | Unknown |
| AIO21889.1 | hypothetical protein KQ76_11750 | SAS081 | 0.34 | 0.44 | | Cytoplasmic |
| AIO21919.1 | urease subunit gamma | ureA SA2082 | 0.35 | 0.45 | Metabolic pathways, Microbial metabolism in diverse environments, Purine metabolism, Arginine biosynthesis | Cytoplasmic |
| AIO19988.1 | alpha/beta hydrolase | SACOL0391 | 0.32 | 0.42 | | Cytoplasmic |
| AIO22336.1 | pyrrolidone-carboxylate peptidase | pcp SACOL2714 | 0.32 | 0.41 | | Cytoplasmic |
| AIO22042.1 | glutathione S-transferase | SACOL2402 | 0.29 | 0.40 | | Unknown |
| AIO21116.1 | gamma-hemolysin subunit A | hlgC SA2208 | 0.35 | 0.48 | | Extracellular |

| | | | | | | |
|---|---|---|---|---|---|---|
| AIO22074.1 | glycerate kinase | SA2220 | 0.24 | 0.34 | Metabolic pathways, Microbial metabolism in diverse environments, Biosynthesis of antibiotics, Glycerolipid metabolism, lyoxylate and dicarboxylate metabolism, Glycine, serine and threonine metabolism | Cytoplasmic |
| AIO21505.1 | serine protease | splF SA1627 | 0.25 | 0.35 | Quorum sensing | Extracellular |
| AIO20749.1 | succinate dehydrogenase | sdhB | 0.28 | 0.41 | Metabolic pathways, Microbial metabolism in diverse environments, Biosynthesis of secondary metabolites, Biosynthesis of antibiotics, Carbon metabolism, Citrate cycle (TCA cycle), Butanoate metabolism, Oxidative phosphorylation | Cytoplasmic |
| AIO21157.1 | replication protein | | 0.31 | 0.47 | | Cytoplasmic |
| AIO21221.1 | acetyl-CoA carboxylase | accB | 0.23 | 0.40 | Metabolic pathways, Microbial metabolism in diverse environments, Biosynthesis of secondary metabolites, Biosynthesis of antibiotics, Carbon metabolism, Fatty acid metabolism, Propanoate metabolism, Pyruvate metabolism, Fatty acid biosynthesis | Cytoplasmic |

**Table S3:** The list of significantly upregulated proteins in hydrated biofilm in comparison to DSB ($p<0.05$). * CM denotes Cytoplasmic Membrane.

| Accession ID | Protein Name | Gene Name | Fold Change | Protein Pathways | Subcellular Localization |
|---|---|---|---|---|---|
| AIO21660.1 | DNA-binding protein | | 2.968 | | Cytoplasmic |
| AIO20800.1 | phosphoribosyl transferase | pyrR SA1041 | 2.527 | Metabolic pathways, Pyrimidine metabolism | Cytoplasmic |
| AIO22003.1 | L-lactate permease | SACOL2363 | 2.495 | | CM* |
| AIO20486.1 | hypothetical protein KQ76_04110 | SA0772 | 2.359 | | Unknown |
| AIO21982.1 | hypothetical protein KQ76_12230 | SACOL2344 | 2.319 | | Cytoplasmic |
| AIO20657.1 | cytochrome C oxidase subunit III | qoxC SA0911 | 2.312 | Metabolic pathways, Oxidative phosphorylation | CM |
| AIO22274.1 | tributyrin esterase | SA2422 | 2.304 | | Unknown |
| AIO21564.1 | general stress protein | SA1692 | 2.294 | | Cytoplasmic |

| | | | | | |
|---|---|---|---|---|---|
| AIO20599.1 | peptide chain release factor 3 | prfC SACOL1025 | 2.272 | | Cytoplasmic |
| AIO20680.1 | hypothetical protein KQ76_05130 | SA0933 | 2.177 | | Unknown |
| AIO21790.1 | glucosamine--fructose-6-phosphate aminotransferase | glmS SACOL2145 | 2.130 | Metabolic pathways, Biosynthesis of antibiotics, Alanine, aspartate and glutamate metabolism, Amino sugar and nucleotide sugar metabolism | Cytoplasmic |
| AIO21830.1 | galactose-6-phosphate isomerase | lacA SA1997 | 2.127 | Metabolic pathways, Galactose metabolism | Unknown |
| AIO22037.1 | nitrite reductase | nasD | 2.078 | Microbial metabolism in diverse environments, Nitrogen metabolism | Cytoplasmic |
| AIO21798.1 | arginase | arg SA1968 | 2.039 | Metabolic pathways, Biosynthesis of secondary metabolites, Biosynthesis of antibiotics, Biosynthesis of amino acids, Arginine biosynthesis, Arginine and proline metabolism | Cytoplasmic |

| | | | | | |
|---|---|---|---|---|---|
| AIO20370.1 | transporter associated domain protein | SA0657 | 2.038 | | CM |
| AIO21892.1 | aminoacyltransferase | femX fmhB SA2057 | 2.026 | Metabolic pathways, Peptidoglycan biosynthesis | Cytoplasmic |
| AIO21786.1 | lytic regulatory protein | truncated-SA | 2.024 | | CM |
| OOC91313.1 | 5-(carboxyamino)imidazole ribonucleotide mutase | purE SA0916 | 2.007 | Metabolic pathways, Biosynthesis of secondary metabolites, Biosynthesis of antibiotics, Purine metabolism | CM |
| AIO20536.1 | argininosuccinate synthase | argG SA0822 | 2.006 | Metabolic pathways, Biosynthesis of secondary metabolites, Biosynthesis of antibiotics, Biosynthesis of amino acids, Arginine biosynthesis, Alanine, aspartate and glutamate metabolism | Cytoplasmic |

| | | | | | |
|---|---|---|---|---|---|
| AIO20949.1 | threonine synthase | thrC SACOL1363 | 2.004 | Metabolic pathways, Biosynthesis of secondary metabolites, Microbial metabolism in diverse environments, Glycine, serine and threonine metabolism, Vitamin B6 metabolism, Biosynthesis of amino acids | Cytoplasmic |

**Table S4:** The list of significantly upregulated proteins in DSB in comparison to hydrated biofilm ($p<0.05$). * CM denotes Cytoplasmic Membrane.

| **Accession ID** | **Protein Name** | **Gene Name** | **Fold Change** | **Protein Pathways** | **Subcellular Localization** |
|---|---|---|---|---|---|
| OOC91425.1 | serine acetyltransferase | cysE SA0487 | 2.322 | Metabolic pathways, Microbial metabolism in diverse environments, Biosynthesis of secondary metabolites, Cysteine and methionine metabolism, Sulfur metabolism, Biosynthesis of amino acids, Carbon metabolism | Cytoplasmic |
| AIO21414.1 | PTS glucose transporter subunit IIBC | ptaA | 2.318 | Amino sugar and nucleotide sugar metabolism, Phosphotransferase system (PTS) | CM* |
| AIO21824.1 | 6-phospho-beta-galactosidase | lacG SA1991 | 2.196 | Metabolic pathways, Galactose metabolism | Cytoplasmic |
| AIO21431.1 | UDP-N-acetylmuramate--alanine ligase | murC SACOL1790 | 2.174 | Metabolic pathways, D-Glutamine and D-glutamate metabolism, Peptidoglycan biosynthesis | Cytoplasmic |

| | | | | | |
|---|---|---|---|---|---|
| AIO20705.1 | inositol monophosphatase | SACOL1116 | 2.128 | Metabolic pathways, Streptomycin biosynthesis, Inositol phosphate metabolism | Cytoplasmic |
| AIO20408.1 | UDP-N-acetylenolpyruvoylglucosamine reductase | murB SA0693 | 2.122 | Metabolic pathways, Amino sugar and nucleotide sugar metabolism, Peptidoglycan biosynthesis | Cytoplasmic |
| AIO22036.1 | nitrite reductase | nasE | 2.091 | Microbial metabolism in diverse environments, Nitrogen metabolism | Cytoplasmic |
| AIO20589.1 | enoyl-ACP reductase | fabI | 2.086 | Metabolic pathways, Biotin metabolism, Fatty acid biosynthesis and metabolism, Biosynthesis of antibiotics | CM |
| AIO20891.1 | recombinase RecA | recA SA1128 | 2.036 | Homologous recombination | Cytoplasmic |
| AIO20673.1 | phosphoribosylamine-glycine ligase | purD SA0926 | 2.011 | Metabolic pathways, Biosynthesis of secondary metabolites, Biosynthesis of antibiotics, Purine metabolism | Cytoplasmic |

**Table S5:** The list of significantly downregulated proteins in hydrated biofilm in comparison to DSB ($p<0.05$). * CM denotes Cytoplasmic Membrane.

| Accession ID | Protein Name | Gene Name | Fold Change | Protein Pathways | Subcellular Localization |
|---|---|---|---|---|---|
| AIO22143.1 | hypothetical protein KQ76_13105 | SACOL2519 | 0.303 | | Cytoplasmic |
| AIO20170.1 | hypothetical protein KQ76_02355 | SA0464 | 0.427 | | Cytoplasmic |
| OOC90925.1 | serine protease splA | splB SA1630 | 0.431 | | Cytoplasmic |
| AIO19949.1 | type VII secretion protein EsaA | esaA SA0272 | 0.432 | | CM* |
| AIO21302.1 | transcription elongation factor GreA | greA SA1438 | 0.435 | | Cytoplasmic |
| AIO20865.1 | ribosome recycling factor | frr SA1102 | 0.435 | | Cytoplasmic |
| AIO21853.1 | 50S ribosomal protein L17 | rplQ SA2022 | 0.436 | Ribosome | Cytoplasmic |
| AIO21993.1 | hemin ABC transporter ATP-binding protein | hrtA SA2149 | 0.448 | ABC transporters | CM |

| | | | | | |
|---|---|---|---|---|---|
| AIO20742.1 | DNA polymerase | SA0990 | 0.451 | | Cytoplasmic |
| AIO19898.1 | uroporphyrin-III methyltransferase | SA0230 | 0.453 | | Unknown |
| AIO20835.1 | acyl carrier protein | acpP hmrB SA1075 | 0.473 | | Cytoplasmic |
| AIO20893.1 | hypothetical protein KQ76_06215 | SACOL1306 | 0.480 | | Cytoplasmic |
| AIO20478.1 | thioredoxin | SA0758 | 0.484 | | Cytoplasmic |
| AIO22260.1 | sulfite reductase [NADPH] flavoprotein alpha-component | SA2413 | 0.486 | Metabolic pathways, Microbial metabolism in diverse environments, Sulfur metabolism | CM |
| AIO20838.1 | cell division protein FtsY | ftsY SACOL1251 | 0.486 | Protein export, Bacterial secretion system, Quorum sensing | CM |
| AIO19963.1 | QueA protein | SACOL0282 | 0.489 | | Cytoplasmic |
| AIO20555.1 | phosphatidylethanolamine-binding protein | SACOL0984 | 0.491 | | Unknown |
| AIO20584.1 | GTP pyrophosphokinase | SA0864 | 0.496 | Purine metabolism | Cytoplasmic |

| | | | | | |
|---|---|---|---|---|---|
| AIO20515.1 | hypothetical protein KQ76_04260 | SACOL0943 | 0.497 | | Cytoplasmic |
| AIO21475.1 | glyoxal reductase | SA1606 | 0.499 | | Cytoplasmic |
| AIO20687.1 | hypothetical protein KQ76_05170 | SA0941 | 0.501 | | Cytoplasmic |
| AIO21223.1 | peptidase M24 | SACOL1588 | 0.505 | | Cytoplasmic |
| AIO22050.1 | amino acid ABC transporter substrate-binding protein | SACOL2412 | 0.508 | ABC transporters | Unknown |
| AIO21432.1 | cell division protein FtsK | SA1562 | 0.510 | | CM |

**Table S6:** The list of significantly downregulated proteins in DSB in comparison to hydrated biofilm ($p<0.05$). * CM denotes Cytoplasmic Membrane.

| Accession ID | Protein Name | Gene Name | Fold Change | Protein Pathways | Subcellular Localization |
|---|---|---|---|---|---|
| AIO20286.1 | recombinase | SA0577 | 0.388 | | Cytoplasmic |
| AIO20702.1 | membrane protein | SACOL1113 | 0.409 | | CM* |
| AIO21531.1 | 3'-5' exonuclease | cbf1 | 0.441 | | Cytoplasmic |
| AIO20231.1 | glycosyl transferase family 1 | SACOL0612 | 0.463 | | Cytoplasmic |
| AIO21551.1 | thiol peroxidase | SA1680 | 0.467 | | Cell-wall |
| AIO20579.1 | oligopeptidase PepB | SA0859 | 0.477 | | Cytoplasmic |
| AIO21461.1 | pyridine nucleotide-disulfide oxidoreductase | SACOL1821 | 0.493 | | Cytoplasmic |
| AIO20197.1 | ribonuclease III | mrnC SA0489 | 0.496 | | Cytoplasmic |

| | | | | | |
|---|---|---|---|---|---|
| AIO21408.1 | glycerophosphodiester phosphodiesterase | SACOL1770 | 0.497 | Glycerophospholipid metabolism | Cytoplasmic |
| AIO21699.1 | RNA polymerase sigma factor SigB | sigB | 0.509 | | Cytoplasmic |
| AIO20740.1 | cell division protein ZapA | SA0988 | 0.509 | | Cytoplasmic |

**Table S7:** Relative correlation of selected qPCR gene expression data to TMT protein expression data (exclusive and common) in hydrated biofilm and DSB compared with planktonic. *FC denotes fold change.

| | | | TMT | | RT-qPCR | |
|---|---|---|---|---|---|---|
| **Accession ID** | **Protein Name** | **Gene Name** | **Hydrated biofilm (FC)*** | **DSB (FC)*** | **Hydrated biofilm (FC)*** | **DSB (FC)*** |
| AIO21431.1 | UDP-N-acetylmuramate--alanine ligase | *MurC* | | 2.174 | | 0.206 |
| AIO20408.1 | UDP-N-acetylenolpyruvoylglucosamine reductase | *MurB* | | 2.122 | | 0.668 |
| AIO20645.1 | glutamyl endopeptidase | *SspA* | 0.149 | 0.144 | 0.104 | 0.083 |
| AIO20163.1 | ribose-phosphate pyrophosphokinase | *Prs* | 2.814 | 2.785 | 3.94 | 0.632 |
| AIO20712.1 | pyruvate carboxylase | *Pyc* | | 0.448 | | 0.837 |
| AIO20749.1 | Succinate dehydrogenase | *SdhB* | 0.283 | 0.406 | 3.30 | 1.313 |

**Table S8:** Primers used in this study

| Gene name | Primer name | Primer sequence (5'-3') | Position in gene | PCR size (bp) | Ta (°C) |
|---|---|---|---|---|---|
| *16s rRNA* | Staphy_16sF | GGCGAAGGCGRCTTTCTGG | 704-720 | 95bp | 55 |
| | Staphy_16sR | CGTTTACGGCGTGGACT | 782-798 | | |
| *MucC* | murC F | TAATTGGTGATGGCACAGG | | | |
| | murC R | GTCATAATTGCGTAATCAGG | 395-506 | 112 | 50 |
| *MurB* | murB F | AGGTGGTAATGCCGACTTT | | | |
| | murB R | TAATTACAATGCCGCGAATA | 99-253 | 155 | 55 |
| *SspA* | sspA F | CAGCGACACTTGTGAGTTC | | | |
| | sspA R | TCTGCGTTTGTTGTGGAT | 53-130 | 109 | 55 |
| *Prs* | prs F | TCAGGACCGGCTAAAGAAC | | | |
| | prs R | AGCTTGTGCGATTAAACCAG | 784-918 | 55 | 55 |

| | | | | | |
|---|---|---|---|---|---|
| *Pyc* | pyc F | TCGTACAACGGCTATCAAGG | | | |
| | pyc R | AATCATTAGCGGGAAACCAG | 366-480 | 60 | 60 |
| *SdhB* | sdhB F | GCCACGTATGCCAGAGAAA | | | |
| | sdhB R | CGAACTTGCGAGATTGCTT | 462-599 | 60 | 60 |
